# *In Vitro* Demonstration of Focused Ultrasound Thrombolysis Using Bifrequency Excitation

**DOI:** 10.1155/2014/518787

**Published:** 2014-08-27

**Authors:** Izella Saletes, Bruno Gilles, Vincent Auboiroux, Nadia Bendridi, Rares Salomir, Jean-Christophe Béra

**Affiliations:** ^1^Inserm, U1032, LabTau and Université de Lyon, 69003 Lyon, France; ^2^Université Lyon 1, 69003 Lyon, France; ^3^Faculty of Medicine, University of Geneva, 1211 Geneva, Switzerland; ^4^Clinatec/LETI/CEA, 38054 Grenoble, France; ^5^Radiology Department, University Hospitals of Geneva, 1211 Geneva, Switzerland; ^6^Inserm, ADR 05 Rhône-Alpes, Auvergne, 69500 Bron, France

## Abstract

Focused ultrasound involving inertial cavitation has been shown to be an efficient method to induce thrombolysis without any pharmacological agent. However, further investigation of the mechanisms involved and further optimization of the process are still required. The present work aims at studying the relevance of a bifrequency excitation compared to a classical monofrequency excitation to achieve thrombolysis without any pharmacological agent. *In vitro* human blood clots were placed at the focus of a piezoelectric transducer. Efficiency of the thrombolysis was assessed by weighing each clot before and after sonication. The efficiencies of mono- (550 kHz) and bifrequency (535 and 565 kHz) excitations were compared for peak power ranging from 70 W to 220 W. The thrombolysis efficiency appears to be correlated to the inertial cavitation activity quantified by passive acoustic listening. In the conditions of the experiment, the power needed to achieve 80% of thrombolysis with a monofrequency excitation is reduced by the half with a bifrequency excitation. The thermal effects of bifrequency and monofrequency excitations, studied using MR thermometry measurements in turkey muscle samples where no cavitation occurred, did not show any difference between both types of excitations when using the same power level.

## 1. Introduction

Blood clot formation is a natural mechanism to prevent and stop bleeding, but it may lead to many vascular diseases like deep vein thrombosis or pulmonary embolism. Currently, thrombosis treatments correspond to two types of approaches that can be combined: surgical and other interventional techniques on one hand and drug based methods on the other hand [1–3]. Surgical methods are strongly invasive and not usable for patients who are usually already weak. Endovascular techniques are less invasive, but they nevertheless require adequate technical centres with qualified staff. In both cases the setting up of the treatment is long and may induce revascularization delays potentially associated with irreversible lesions on tissue. Thrombolytic drugs are usually used in the treatment of thrombosis: thanks to their large availability and their ease of use, they have greatly enhanced treatments of vascular diseases, but they still present a number of major drawbacks [4]: due to their nonselective action when they are injected in general bloodstream, they carry a significant haemorrhagic risk, and their administration is restricted by a lot of contraindications. Recent thrombolysis methods were proposed to combine thrombolytic agents with percutaneous techniques in order to get more selective treatments [5, 6], but haemorrhagic risks remain and constraints related to catheterization still require adequate technical centres.

Considering the limitations of these treatments, the use of ultrasound for thrombolysis has been foreseen as a promising technique especially when combined with fibrinolytic agents [7]. Ultrasound can be applied either using a catheter to locally sonicate blood clots [8–10] or externally. Catheter-based techniques enable rapid clot lysis, but they have the drawbacks of interventional techniques mentioned above. In the hope of reducing invasiveness, many studies focused on the use of transcutaneous ultrasound. In particular, a number of works have shown that low-intensity ultrasound was able to increase the activity of fibrinolytic agents [11–16], which enables reducing the drug quantity required for thrombolysis. Nevertheless, haemorrhagic risk associated with the use of thrombolytic drugs is still present, and resisting clots were also observed [17]. Transcutaneous ultrasound thrombolysis could be also achieved without any pharmacological agent using focused shock waves or high intensity ultrasound [18, 19] and recently received increasing attention with studies showing that transcutaneous focused ultrasound can achieve clot lysis within minutes both* in vitro* and* in vivo* [20–23]. In those works, cavitation appeared to play a major role in clot disruption, even if mechanisms involved in the process still have to be clarified.

Stimulation of the inertial cavitation activity can be achieved using a focused ultrasound excitation including two neighbouring frequencies instead of a monofrequency wave: this type of excitation enables, in some configurations, lowering of the inertial cavitation threshold [24] and an increase in the cavitation activity on a target of controlled roughness [25].

Such a stimulation of the cavitation activity could be an advantage for ultrasound thrombolysis, but because of higher pressure amplitudes involved for a bifrequency excitation at a given intensity, increased heating of tissue due to nonlinear propagation effects could also occur [26], which would be a drawback of the method. The present study aims at comparing mono- and bifrequency excitations both in terms of thrombolytic efficiency for an* in vitro* blood clot model and in terms of heating of tissue in the focal region. In a first set of experiments, passive ultrasound monitoring of the cavitation activity during thrombolysis is processed, and in a second set of experiments, MR imaging is used to characterize the temperature rise caused by both types of excitation and to dynamically visualize the clot disruption process. A single sonication configuration is used, with peak amplitudes comparable to the one successfully used by Maxwell et al. [20] to achieve ultrasound thrombolysis, but with increased duty cycle in order to achieve a better characterization of the thermal effects in the case of a bifrequency excitation at high exposure levels, when compared to a monofrequency excitation.

## 2. Materials and Methods

### 2.1. Ultrasound Generation

Acoustic excitations were generated by a focused spherical piezoelectric transducer (focal length: 100 mm, aperture diameter: 100 mm). The resonant frequency of the whole emission line, including a generator (AFG3102, Tektronix, USA), a power amplifier (1000 W, 0.1–6 MHz, Adece, France), and the transducer (Imasonic, France), was 550 kHz. The −3 dB focal volume at the resonant frequency was a 20 mm long and 3 mm wide ellipsoid. The transducer efficiency at the resonant frequency, measured using a radiation force balance method, was 84%.

Two types of pulsed-wave excitation were used, according to [25]:

- a monofrequency excitation which consisted in a pulsed-wave signal of a pure sine wave at frequency *f*_0_ = 550 kHz,
- a bifrequency excitation which consisted in a pulsed-wave signal made of the sum of two sine waves of slightly different frequencies, *f*_1_ = 535 kHz and *f*_2_ = 565 kHz, and of the same pressure amplitude.

In both cases, the pulse duration was 27 ms, the duty cycle was 1 : 10, and the total treatment duration was 5 minutes. The exposure level was expressed in terms of the acoustic power that ranged from *P*_ac_ = 70 W to *P*_ac_ = 220 W for both types of excitation. In order to characterize the peak negative and positive pressures obtained in that range of excitation level, and the corresponding spatial-peak pulse-average intensities, a calibration was done using a hydrophone (Müller-Platte Needle Probe, Müller Instruments, Germany) placed at the focal point of the transducer in the absence of any clot. In order to avoid cavitation at high excitation levels, shorter pulses were used for this calibration process: 20 *μ*s pulses for monofrequency excitation and 70 *μ*s pulses for bifrequency excitation. Results are given in Table 1.

**Figure 1:**
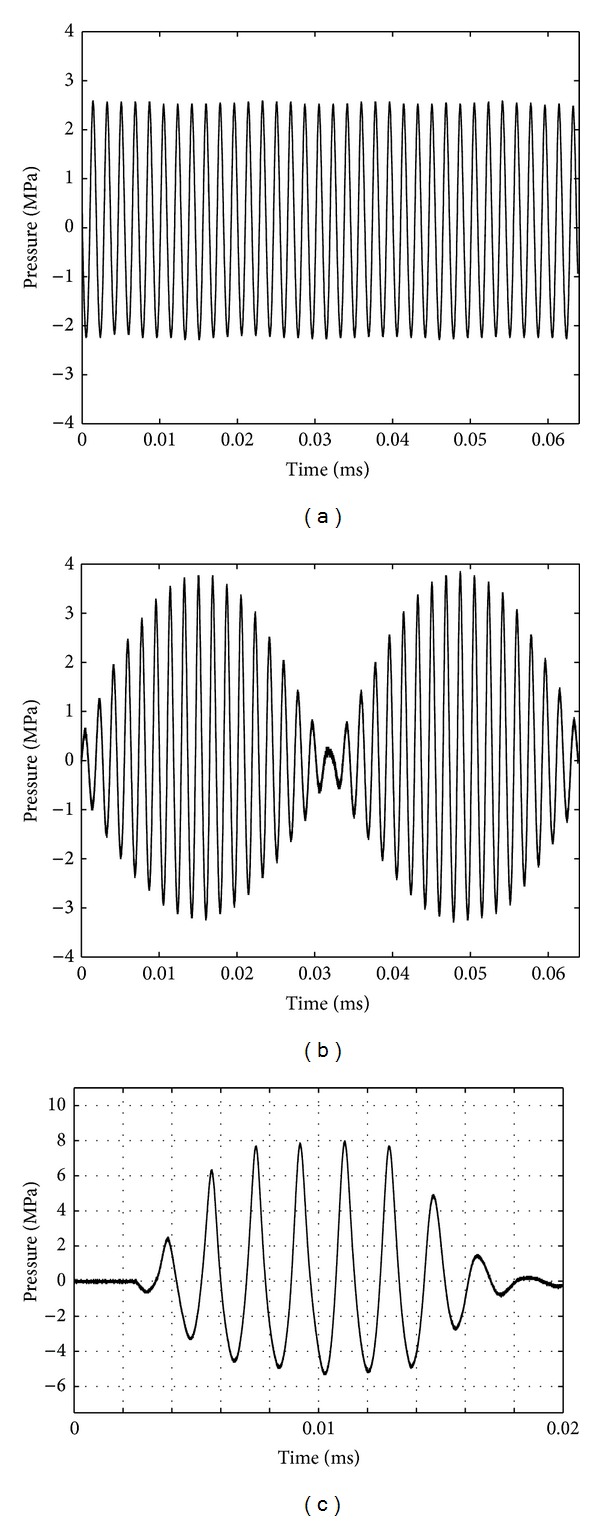
Pressure waveforms for (a) mono- and (b) bifrequency excitations, measured at the focal point of the transducer at a power *P*_ac_ = 25 W. (c) illustrates a pressure waveform measured during the calibration process at *P*_ac_ = 165 W. A short pulse was applied to avoid cavitation on the hydrophone.

Examples of pressure waveforms measured at the focal point of the transducer are shown in Figure 1 at a power *P*_ac_ = 25 W, for which no cavitation occurred, and at *P*_ac_ = 165 W using a short pulse to avoid cavitation at this exposure level. It has to be noticed that, at a given power, the peak pressure of a bifrequency signal is $\sqrt{2}$ times higher than the one of a monofrequency signal, as can be seen on peak pressures of the signals in Figure 1.

A duty cycle as high as 1 : 10 was chosen to stimulate heating in the focal region, in order to characterize, as precisely as possible, the spatial distribution of the bifrequency pressure field in tissue at high amplitudes, and to compare temperature rise for both types of excitation at a given power. This aimed at evaluating the nonlinear propagation effects associated with the bifrequency waveforms by measuring the nonlinear increase in temperature rise—compared to a monofrequency wave of the same power—due to the higher peak pressure being involved in the bifrequency waveforms.

### 2.2. Thrombolysis with Passive Listening of Inertial Cavitation Activity

**Figure 2:**
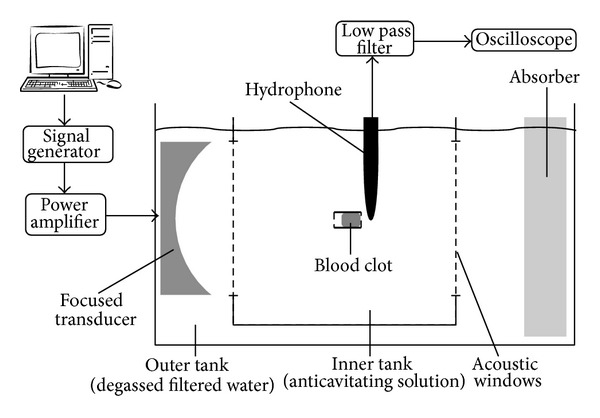
Sketch of the experimental setup.

The thrombolysis experiments were carried out in a double-tank sketched in Figure 2. The outer tank (520 × 270 × 230 mm (*L* × *W* × *H*)) was filled with degassed filtered water. To enable good transmission and avoid acoustic reflections, two acoustic windows were pierced in the inner tank (200 × 200 × 215 mm (*L* × *W* × *H*)), and an acoustic absorber consisting of a paraffin block was placed at the back of the outer tank. The blood clot samples (cf. below) were placed in PVC tubes (ID = 10 mm, OD = 12 mm, and *H* = 15 mm) at the focus of the transducer. In order to prevent cavitation occurring on the front side of the PVC tube outside from the tube, the smaller tank was filled with a solution of Polyvinylpyrrolidone (PVP) diluted at 30 g · L^−1^ [27].

In order to quantify the activity of inertial cavitation during sonication, a low-frequency hydrophone (Reson TC4034, Denmark, bandwidth [1; 500 kHz]) was placed behind the PVC tube, in the axial plane. The acoustic centre of the hydrophone was positioned 1.2 cm behind the PVC tube and 1 cm above the acoustical axis. The signal recorded by the hydrophone was low pass filtered using a 4th order filter with a cut-off frequency of 400 kHz, before being digitized using a numerical oscilloscope (Wavesurfer 24XS, Lecroy, USA). For each 300 s experiment (cf.  Section 2.1), 19 signal samples of 27 ms were saved, one every 16 s. For each sample, the Inertial Cavitation Index (ICI) was defined as the average inertial cavitation activity computed every 1.4 ms according to the method detailed in [25]. ICI was recorded as a function of time. The ICI values were time-averaged to get 〈ICI〉 which was an estimation of the mean inertial cavitation activity during the whole experiment. The plot of thrombolysis efficiency as a function of 〈ICI〉 enabled analysing the correlation between cavitation activity and thrombolysis.

Clots were formed from human blood obtained from anonymous, healthy volunteers, under the agreement of the “Etablissement Français du Sang” (French Blood Establishment) and the French Institute of Health and Medical Research (Inserm). Once the blood sample had been taken, it was kept at 4°C during the serological control (between 12 h and 24 h). Then, 100 mL of blood was mixed, at 37°C, with 4 mM of CaCl_2_ and 5  UNIH of human thrombin, which was stabilized with a bovine albumin solution (diluted in physiological serum at 1%). The mix was poured into a 100 mL Petri dish (*⌀*100 mm × 12 mm), which was incubated at 37°C for 5 min⁡ during coagulation process. Once coagulated, the blood was preserved at 4°C in the closed Petri dish during three days in order to ensure clot retraction [28–31]. It was then used for the experiments within three days.

To carry out thrombolysis experiments, 10 mm in diameter and 10 mm in height cylindrical blood clots (volume = 785 mm^3^) were taken from Petri dish using a die-cutter. Before sonication, a blood clot was rinsed using physiological serum on a 25 *μ*m filter and weighed, providing the initial clot mass *m*_clot_. The average weight of clots was 724 mg with standard deviation of 120 mg. The blood clot was then placed in the PVC tube; the remaining volume was filled with physiological serum and the tube was closed at both ends with latex membranes. For sonication, the tube was then placed at the focus of the transducer, the liquid part being placed in front of the transducer and the clot being in contact with the back of the tube (cf. Figure 2). The focal point of the transducer was placed 8 mm deep after the front interface of the clot. After sonication, the PVC tube was drained and rinsed on a 25 *μ*m filter and the residual mass (*m*_residual_) was weighed.

The thrombolysis efficiency was defined as the percentage of lysed clot:, (1 − *m*_residual_/*m*_clot_) × 100. For each intensity and each type of excitation, 5 to 7 clots were sonicated; the total number of sonicated clots was 134. Throughout the study, nonsonicated clots were weighed before and after the above-mentioned procedure and constituted a control group of 18 samples.

The values were summarized as mean. The relationship between both excitations was examined by the Mann-Whitney test. *P* < 0.05 and *P* < 0.001 indicated statistically significant (∗) and highly significant difference (∗∗), respectively.

### 2.3. Mono- versus Bifrequency Sonication Follow-Up Using MR Imaging

In order to further characterize the effects associated with both types of excitation, complementary experiments combining sonication with fast MR acquisitions were performed. A MRI compatible version of the setup described in Section 2.2 enabled the use of a gradient echo-based sequence (T2*-weighted) for two different sets of experiments: (1) dynamical imaging of lysis process operated on large cylindrical specimens of blood clots (30 mm in diameter, 30 mm in height); (2) a comparison of the temperature profiles induced by both types of excitation using sensitivity of the gradient echo sequence to temperature elevation (with proton resonance frequency shift effect). Passive detection of cavitation using a hydrophone was not available with MR imaging.

All MR imaging experiments were performed on a 3.0 T clinical MR-scanner (TIM Trio, Siemens, Germany) using a standard 4-channel flex coil for MR signal acquisition. Measurement of temperature elevation during sonication was performed using MR thermometry based on PRFS effect [32]. A segmented gradient-recalled echo-planar imaging (GRE-EPI) sequence was used to provide PRFS-sensitive images. The main imaging acquisition parameters were *TR* = 45 ms, *TE* = 6.3 ms, and voxel size = 0.75 × 0.75 × 5 mm (256 × 256 matrix). The acquisition time per measurement was 5 s for two orthogonal interleaved slices (centred on the focal point each and aligned with the revolution axis of the transducer). RF saturation slabs were prescribed laterally from the target in order to reduce the MR effects of HIFU-induced streaming in the water tank. Due to the limitations of the PRFS method, temperature profiles could not be measured during thrombolysis within the blood clot, mainly because of the presence of the moving liquid phase in the lysed region of the thrombus around the focal point of the transducer, as the drilled hole was getting filled with water. Beside the local macroscopic motion of MR observed protons, dynamic changes in the bulk magnetic susceptibility are expected in the process of clot dissolution. The comparison of the temperature profiles with monofrequency and bifrequency sonications was therefore performed in* ex vivo* turkey muscle.

The sample to be sonicated, either blood clot or turkey muscle, was placed in a PVC tube (ID = 30 mm, OD = 32 mm, and *H* = 70 mm) which was closed at both ends with condoms. Muscle samples completely filled the PVC tubes, while tubes containing the blood clots were filled with physiological serum on the side proximal to the transducer. The tube was positioned in order to get the focal point 5 mm deep after the front interface of the clot. For muscle samples, the focus was positioned at the center of the sample, 35 mm away from the front interface. The tubes were immersed in a degassed water tank that ensured acoustic coupling and isothermal conditions at specimen edges.

Temperature maps were calculated offline on a slice-per-slice basis using the reference-free method proposed by [33]. This approach was chosen as it intrinsically removes the need of correction for *B*_0_ drift and phase shift due to surrounding water streaming. Although this streaming is spatially located outside the region of interest (ROI), it induces “ghosting-” like phase shift effects also inside the ROI via the *k*-space contamination, which further prevents the use of time referenced PRFS thermometry. The Dirichlet domain was defined to be circular and as large as possible within the* ex vivo* tissue (20 mm in diameter). Note that increasing the domain size helps in reducing the risk of thermal contamination of its border and thus the subsequent risk of underestimation of the temperature elevation. The voxels from the domain border having an SNR inferior to 15, if any, were removed from calculation and a closed border was regenerated using the iterative harmonic interpolation described in [33]. Baseline MR acquisition without sonication was performed in order to calculate the intrinsic standard deviation of the MR thermometry (as described in [34]). The pixelwise standard deviation was evaluated to be 0.7°C.

## 3. Results

### 3.1. Thrombolysis Efficiency

**Figure 3:**
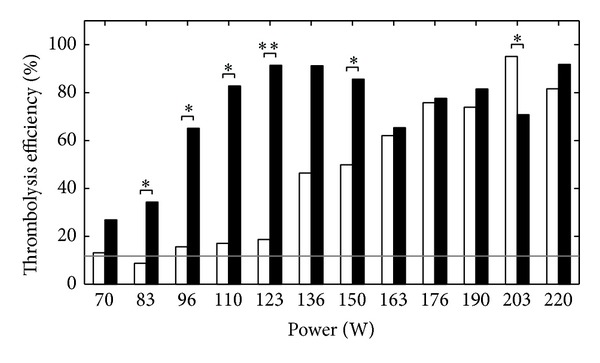
Thrombolysis efficiency as a function of the power (*P*_ac_), for a monofrequency excitation (white bars) and a bifrequency excitation (black bars). *P < 0.05 and **P < 0.001. The grey line corresponds to the baseline thrombolysis of the nonsonicated control group (12 ± 7% (mean ± standard deviation)).

Thrombolysis efficiencies were measured for both types of excitation and for different power values (*P*_ac_), and the results are summarized in Figure 3. The baseline thrombolysis for the nonsonicated control group of 18 clots was 12 ± 7% (mean ± standard deviation). Therefore we shall consider the ultrasound-induced thrombolysis as efficient only for clot dissolution above 19%. For the monofrequency excitation, no thrombolysis was detected for power values lower than 130 W. Beyond this exposure level, the thrombolysis efficiency increased to achieve a maximum value of 95 ± 8% for a power of 200 W.

In the case of the bifrequency excitation, a thrombolytic effect was observed even for the lowest exposure level (70 W). The efficiency achieved a maximum value of 91 ± 10% at 123 W. For larger exposure levels, the thrombolysis efficiency remained larger than 70% despite an irregular behavior, mainly due to some screening of the acoustic wave by cavitation bubbles produced upstream the PVC tube at such powers, with a bifrequency excitation.

For powers below 130 W, the bifrequency efficiency was several times larger than the monofrequency one. In particular, at 120 W, the bifrequency efficiency was 90%, while there was no significant thrombolysis with the monofrequency excitation.

### 3.2. Correlation between Inertial Cavitation Activity and Thrombolysis Efficiency

**Figure 4:**
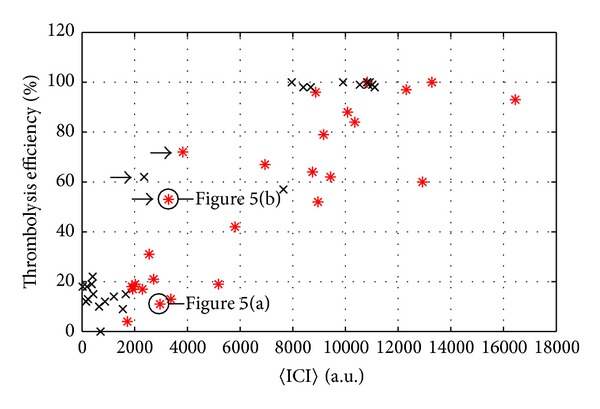
Thrombolysis efficiency as a function of average Inertial Cavitation Index 〈ICI〉, for a monofrequency excitation (black crosses) and a bifrequency excitation (red asterisks).

The thrombolysis efficiency is plotted in Figure 4 as a function of 〈ICI〉. Despite some dispersion, especially observed for intermediate 〈ICI〉 values, one can note an overall increase in thrombolysis efficiency for increasing inertial cavitation activity, with no thrombolysis observed for 〈ICI〉 below 2000 a.u. For 〈ICI〉 beyond 6000 a.u., the thrombolysis efficiency was always larger than 50%.

**Figure 5:**
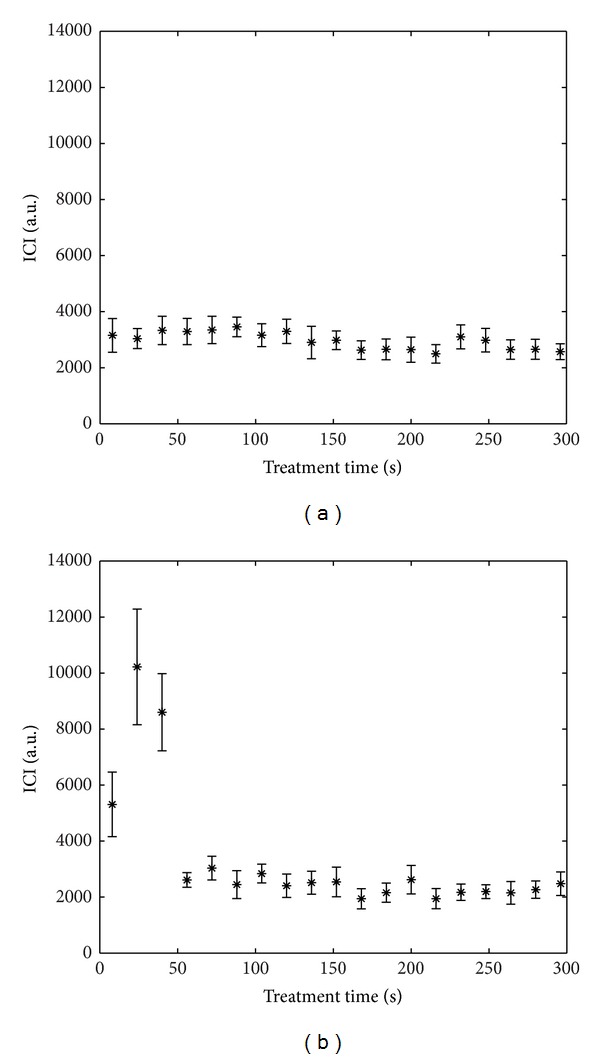
Examples of time evolution of the Inertial Cavitation Index (ICI) for two samples (bifrequency excitation, *P*_ac_ = 110 W). Error bars show standard deviation. 〈ICI〉 on total treatment duration is about 3000 a.u. for both cases. The corresponding thrombolysis efficiency is 11% for (a) and 53% for (b).

In order to explain the strong dispersion observed for 〈ICI〉 between 2000 and 6000 a.u., the time fluctuations of ICI have to be considered; Figures 5(a) and 5(b) show the time evolution of ICI for two realizations corresponding to an identical bifrequency treatment at 110 W. In both cases, 〈ICI〉 was about 3000 a.u. while the corresponding thrombolysis efficiencies were, respectively, 11% for Figure 5(a) and 53% for Figure 5(b). However, ICI time evolutions were very different from one case to the other: in Figure 5(a) ICI was roughly constant during the treatment, whereas it was above 5000 a.u. during the first 50 s in Figure 5(b). In the latter case, the short duration where ICI > 5000 a.u. appeared to be sufficient to lyse 53% of the blood clot whereas the constant ICI≃3000 a.u. of Figure 5(a) induced no lysis. Similar time behaviours, regardless of the excitation, could be observed for the realizations corresponding to the points marked with an arrow in Figure 4.

### 3.3. MR Monitoring of Mechanical and Thermal Effects

**Figure 6:**
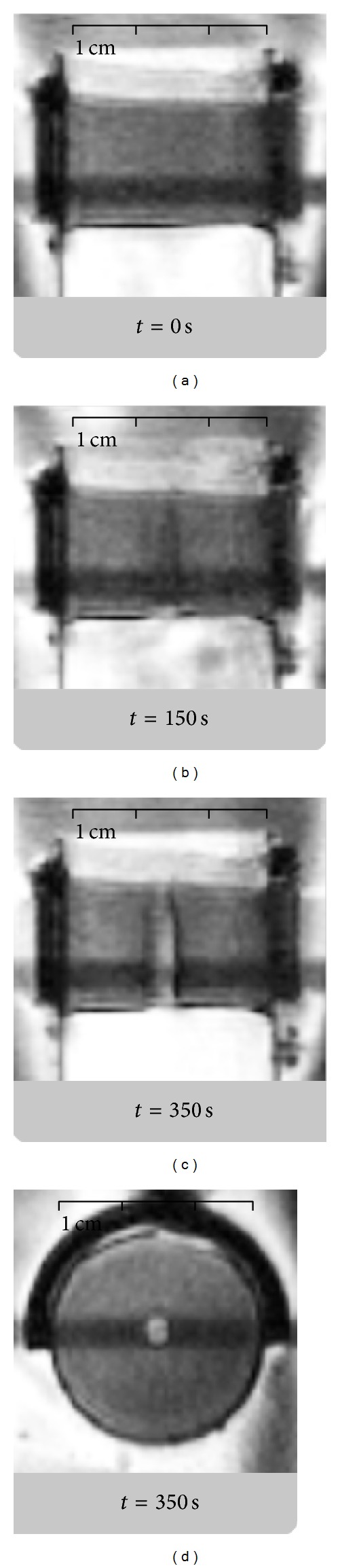
MR imaging of a blood clot during sonolysis (bifrequency excitation, *P*_ac_ = 40 W) before (a), during (b), and after (c and d) the complete drilling of the sample. (a, b, and c) Acquisitions are performed in coronal plane aligned with the acoustical axis while (d) corresponds to a through-focus axial plane (orthogonal to the acoustical axis). The hypointense bands correspond to MR signal saturation at interleaved slice crossing.

Figure 6 shows the MR magnitude images obtained during sonication of a blood clot sample. The progression of blood clot dissolution during the procedure was clearly visible as an increase in MR signal in the hole drilled by ultrasound, once it was filled with physiological serum.

**Figure 7:**
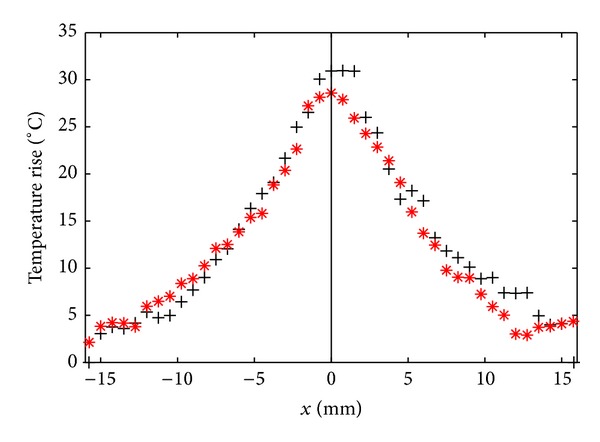
Temperature rise along transverse axis for a monofrequency excitation (black crosses) and a bifrequency excitation (red asterisks), after 300 s of pulsed sonication 10% duty cycle. The power is equal to 200 W for both cases. The origin represents the intersection with the acoustical axis of the transducer.

The same setup, using turkey muscle samples instead of blood clots, enabled the comparison of thermal effects for mono- and bifrequency excitations. Typical temperature profiles, along transverse axis in the focal plane at the end of the sonication, are shown in Figure 7 for a power of 200 W. The profile was averaged over 5 samples for each type of excitation, and the intersample standard deviation, omitted from the graph for clarity reasons, was equal to 3.9°C and 4.8°C on average over the whole profile for bifrequency and monofrequency excitations, respectively. For any exposure level, the profiles were the same for both types of excitation and the maximal temperature rise was achieved at the focus.

**Figure 8:**
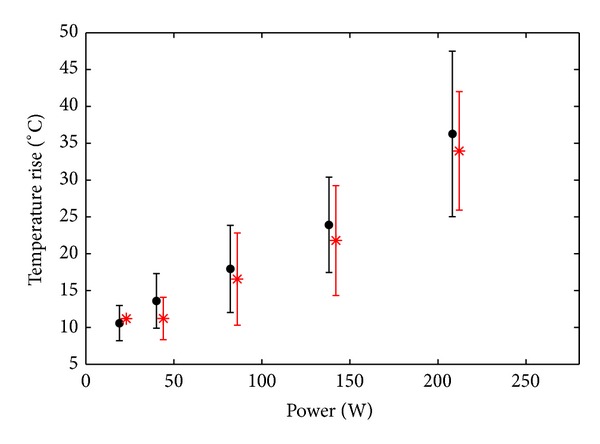
Average temperature rise at the focus as a function of the applied power, for a monofrequency excitation (black discs) and a bifrequency excitation (red asterisks), after 300 s of pulsed sonication 10% duty cycle. Error bars show intersample standard deviation.

Figure 8 shows the average temperature rise reached in muscle samples at the focus for both types of excitation as a function of the power (*P*_ac_). Regardless of the type of excitation (mono- or bifrequency) the local heating patterns seen by MR thermometry in* ex vivo* muscle had very similar behavior as a function of the applied power.

## 4. Discussion

It has been proven that transcutaneous ultrasound alone can induce thrombolysis through the development of cavitation activity both* in vitro* and* in vivo* [20–23], and it has been demonstrated that inertial cavitation plays a major role in the process. Nevertheless, optimizing treatment strategies is still needed to avoid undesirable effects such as inducing lesions to surrounding healthy tissue or tearing off large debris during lysis that could induce embolisation.

From that perspective, a better understanding of the mechanisms involved is a key point in the development of such techniques. Our results concerning passive listening of inertial cavitation during* in vitro* blood clot sonication clearly demonstrated the correlation between inertial cavitation activity and thrombolysis efficiency. But this correlation did not concern the average value over the whole treatment duration. To be efficient for pure ultrasound thrombolysis, the inertial cavitation activity must be quite intense. If the ICI was below 3000 a.u., there was no thrombolysis. Beyond this onset threshold value, the thrombolysis of the clot can be complete in less than 300 s.

In a previous study [25], it was shown that a bifrequency excitation is an efficient means to lower cavitation thresholds when they correspond to high acoustic intensities and to stimulate cavitation activity beyond threshold. In the present study, the results show that the use of a bifrequency excitation drastically enhances thrombolysis efficiency in the intensity range where a monofrequency excitation is poorly efficient. This is notably the case below 120 W, and at this exposure level, the bifrequency thrombolysis efficiency achieved 90% while there was no significant thrombolysis with the monofrequency excitation. A consequence is that the power needed to obtain 80% of thrombolysis efficiency can be reduced by half (200 W with the monofrequency excitation against 110 W with the bifrequency excitation).

In [25], the bifrequency excitation was able to lower the inertial cavitation threshold only for spatial-peak pulse-average (*I*_sppa_) intensities larger than 100 W/cm^2^. It has to be noticed that, in that study, cavitation occurred on a sandpaper target and not on a blood clot: the ratio of the acoustic impedances between water and such targets is much higher than the ratio between water and blood clots; peak pressure at the interface was higher for a given amplitude of the incident wave, resulting in an easier cavitation triggering. As a consequence, in the present study, higher amplitudes had to be used to obtain cavitation: at the lowest exposure level used in this study, the spatial-peak pulse-average intensity reaches 500 W/cm^2^ (see Table 1), and no cavitation was observed for the monofrequency excitation. At such intensities, one can expect a strong stimulation of the cavitation activity due to the use of a bifrequency excitation, according to the results observed in [25]. This results in the higher thrombolysis efficiencies observed in Figure 3 when a bifrequency excitation is used.

Concerning MR results, the choice of a high duty cycle value enabled comparing heat deposition for both types of excitations and to better characterize the behaviour of the bifrequency waveforms at high amplitudes in tissue. Heating induced by ultrasound wave for a given intensity appeared to be the same for both types of excitation: spatial temperature profiles and temperature maxima measured using MR thermometry in muscle samples were basically identical at a given applied *P*_ac_ value. In fact, the use of bifrequency HIFU to enlarge heating lesions in tissue is valid only for amplitudes higher than the cavitation threshold [35], while the constant slopes observed on curves from Figure 8 suggest that no increase of absorption due to the presence of cavitation bubbles occurred during experiments. As for aspects related to the nonlinear propagation of the wave, it was explained in [25] that even for high peak-to-peak amplitude the waveforms should not be shocked because the distance of shock formation is several times higher than the axial length of the −3 dB focal volume (20 mm). The heating patterns obtained in muscle confirmed this aspect and also validated that the acoustic magnitude fields are the same for both types of excitation. For a given acoustic power in liquid, cavitation would be more intense for bifrequency excitation and the nonlinearity would be increased, yielding a larger contribution in terms of nonlinear generation of heat than in structured biological tissue, but for the same thrombolysis efficiency (i.e., the same cavitation activity and the same local nonlinearity due to bubbles) the power required would be lower for bifrequency excitation and so should be the heating.

Obviously, lower duty cycle would be required for* in vivo* thrombolysis applications, in order to reduce time-average intensities and associated temperature rise to safer values. To evaluate the impact this would have on the thrombolysis efficiency, a comparison of our results in the case of a monofrequency excitation to the one obtained in [20, 22], which use 20 to 100 times lower duty cycle values, can be done. Pulse-average acoustic power used in our experiments ranges from 70 to 220 W, with thrombolytic efficiency reaching 50% at 150 W. For* in vitro* results presented in [22] acoustic power ranged from 120 to 185 W, with positive results for thrombolysis beyond 160 W. In [20], exposure levels were expressed in terms of peak pressures. Peak negative pressure was ranging from 2 to 12 MPa, with thrombolytic effects observed beyond 6 MPa. We use 3 to 5.5 MPa, with 50% efficiency achieved at 5 MPa. This shows that, when using a monofrequency waveform, we achieve thrombolysis with pulse-average powers or peak negative pressures similar to the one obtained in those studies which use much lower duty cycle values. Reducing the duty cycle should thus reduce the heat deposition without altering our results for thrombolysis efficiency.

It is also worth noting that MR magnitude data enable online visualization of the blood clot dissolution process (see progressive signal modification in the blood clot, near the focal point in Figure 6). This effect could be used for the online monitoring of treatment progress* in situ*. Nevertheless, a quantitative assessment of the correlation between the variation of MR signal and the structural change in tissue (clot dissolution) is beyond the purpose of this study.

To sum up, the study hereby confirms* in vitro* the interest of a bifrequency excitation compared with a monofrequency one for pure ultrasound thrombolysis. Taking into account the high values of inertial cavitation threshold of blood [36, 37] and nonlinearity parameter of blood and tissue compared to water [38], the bifrequency excitation should be even more efficient for* in vivo* thrombolysis than what is observed for the* in vitro* experiments presented here, since the efficiency of the bifrequency excitation increases with the inertial cavitation threshold of the medium [25].

## 5. Conclusions

The thrombolysis efficiency of a bifrequency excitation consisting in two high neighbouring frequency components has been tested on an* in vitro* blood clot model and compared to the thrombolysis efficiency of a monofrequency excitation of the same ultrasound intensity. The bifrequency excitation could lower by half the power required to achieve a lysis of a 0.8 cm^3^ blood clot in 5 minutes. The phenomenon involved in thrombolysis enhancement is confirmed to be inertial cavitation and the thrombolysis efficiency is shown to depend on the whole cavitation activity history: the cavitation activity has to be larger than a given threshold, but once this condition is fulfilled, the lysis is achieved very rapidly. MR thermometry measurements show that the temperature elevation does not depend on the type of excitation (mono- or bifrequency) by itself but only on the acoustic intensity if cavitation is absent. Concerning the therapeutic application, this study shows that the use of a bifrequency excitation is an interesting way to enhance pure ultrasound thrombolysis by stimulating inertial cavitation activity.

## Figures and Tables

**Table 1:** Peak negative and positive pressures and pulse-average intensities measured at the focal point of the transducer in water for the range of exposure level used in the experiments and for both types of excitations.

Peak pressures		Spatial-peak pulse-average intensity	Total acoustic power
*p* ^−^ (MPa)	*p* ^+^ (MPa)	*I*_sppa_ (W/cm^2^)^a^	*P*_ac_ (W)
Monofrequency excitation			
3	3.6	360	50
4.2	6.2	770	110
5.2	8	1200	165
5.5	9.0	—	220

Bifrequency excitation			
5	7	510	70
6	9	750	110
6.5	10.5	~1000^a^	150
7.5	12.5	—	220

^a^Intensities are estimated by integrating waveforms recorded at the focal point. At *P*_ac_ = 220 W, occurrence of cavitation prevents from giving an accurate estimation of the intensity. Bifrequency waveform at *P*_ac_ = 150 W was also perturbed by cavitation.
